# PEG 6000-Stimulated Drought Stress Improves the Attributes of In Vitro Growth, Steviol Glycosides Production, and Antioxidant Activities in *Stevia rebaudiana* Bertoni

**DOI:** 10.3390/plants9111552

**Published:** 2020-11-12

**Authors:** Muhammad Arslan Ahmad, Rabia Javed, Muhammad Adeel, Muhammad Rizwan, Yuesuo Yang

**Affiliations:** 1 Key Lab of Eco-Restoration of Regional Contaminated Environment, Ministry of Education, Shenyang University, Shenyang 110044, China; arslan.slu@gmail.com; 2 Department of Tissue Engineering, China Medical University, Shenyang 110122, China; 3 Beijing Key Laboratory of Farmland Soil Pollution Prevention and Remediation, College of Resources and Environmental Sciences, China Agricultural University, Beijing 100193, China; Chadeel969@gmail.com; 4 Institute of Soil Science, PMAS Arid Agriculture University Rawalpindi, Rawalpindi 46000, Pakistan; muhammadrizwan@webmail.hzau.edu.cn; 5 Key Lab of Groundwater and Environment, Ministry of Education, Jilin University, Changchun 130021, China

**Keywords:** *Stevia rebaudiana*, steviol glycosides (SGs), antioxidant activities, polyethylene glycol (PEG), drought stress

## Abstract

This study addresses the favourable effects of drought stress imposed by polyethylene glycol (PEG) 6000 on the micropropagated shoots of *Stevia rebaudiana*. Various concentrations, i.e., 0, 0.5, 1, 2, and 4% of PEG 6000 were applied to the nodal shoot explants for four weeks, and the influence produced on shoots growth parameters, bioactive steviol glycosides (rebaudioside A and stevioside), and nonenzymatic antioxidant activities (total phenolic content (TPC), total flavonoid content (TFC), total antioxidant capacity (TAC), total reducing power (TRP) and 1,1-diphenyl-2-picrylhydrazyl(DPPH)-free radical scavenging activity (FRSA)) was elucidated. The significantly highest yield (92.4% direct shoot organogenesis) and secondary metabolites (2.94% Reb A, 2.52% ST, 95.3% DPPH-FRSA, 15.0% TPC, 13.0 µg/mg TFC, 22.3 µg/mg TAC, and 19.8 µg/mg TRP) production in response to abiotic stress elicitors was obtained in Murashige and Skoog (MS) medium treatment provided with 4% of PEG 6000. The overall trend was significant enhancement of growth dynamics and pharmaceutical compounds from control to 4% of PEG 6000 concentration as a defensive response against reactive oxygen species (ROS) produced in excess by water deficit. This is a very promising study to be employed in bioreactors to get markedly enhanced content of compounds of medicinal importance in the pharmaceutical market.

## 1. Introduction

*Stevia rebaudiana*, commonly called “sweet leaf”, is a perennial shrub distributed in South America mainly Brazil and Paraguay [1]. However, it is gaining remarkable significance all over the world because of its high potency sweetness. Rebaudioside A (Reb A) and stevioside (ST) are the major steviol glycosides (SGs) present in the leaf tissues of *Stevia* which are nonmutagenic and nontoxic compounds. The *Stevia* leaves are 300 times sweeter than sugar and have no calories [2,3]. SGs are multifunctional biochemicals and confer antidiabetic, antihypertensive, anti-inflammatory, and anticancerous properties to *Stevia* [4]. Seeds of *S. rebaudiana* show poor germination and the conventional propagation strategies such as cuttings are not effective [5]. Hence, new biotechnological approaches have been adopted for maximum production of *Stevia* progeny and its medicinal components in the minimum period of time [6,7,8,9,10,11,12,13].

Since the yield and production of secondary metabolites by traditional agriculture is not sufficient, plant tissue culture technique has been adapted as an alternative strategy to produce the commercially desired levels of bioactive compounds. Recently, medicinal plants have been exposed to different types of abiotic stresses leading to adaptive physiological changes and metabolic defence response signalling in the formation of secondary metabolites (extracellular or intracellular) to cope with the changing climatic conditions. Drought/dehydration is one of the abiotic stress elicitors against which tolerance can be evaluated using tissue culturing because the stress conditions get easily controlled under in vitro system. Moreover, least variability in plant population is observed in a limited time and space by the efficient tissue culture technique [14,15].

Polyethylene glycol (PEG) having molecular weight of 6000 is a natural polymer that is water-soluble and nonionic [16]. PEG 6000 is found to mimic drought stress and results in lowering of plant’s water potential due to osmotic stress [17]. In the current study, physiological and metabolic changes in response to PEG 6000 were investigated as our understanding of the morphological and biochemical response of *S. rebaudiana* to drought stress is limited. According to the best of our knowledge, there is not a single study in which production of Reb A in response to PEG 6000-associated drought stress imposed under in vitro condition has been elaborated. Previously, Hajihashemi and Geuns [18] evaluated the impact of drought stress on SGs but under greenhouse cultivation system. Moreover, Badran et al. [19] determined the relationship between drought stress and ST content only. However, we conducted this study to explore water-deficit stress induced by PEG 6000 to in vitro grown *S. rebaudiana* shoots and evaluated the resultant effects on the physiology (plant biomass) and biochemistry {(Reb A and ST content) and (antioxidation potential)} of *S. rebaudiana* by combating drought stress. Besides, it is a dire need to obtain sustainable secondary metabolites in bioreactors by modulating tissue culture-grown medicinal plants which can be achieved in the future by following up on this pilot research.

## 2. Results

### 2.1. Evaluation of Growth and Development

The shoots obtained from 4-week-old nodal explants cultivated in different MS growth medium treatments were observed. These treatments involved 0, 0.5, 1.0, 2.0, and 4.0% of PEG 6000 stress. Table 1 shows that the maximum percentage of shooting (~92.4%) of nodal explants was revealed by the treatment containing 4% of PEG 6000 stress. Similarly, the maximum mean length of shoots (5.1 cm), mean number of nodes (5.3), mean number of leaves (17.9), and fresh weight (FW) of shoots (0.52 g) were found under the treatment supplemented with PEG stress of 4% concentration. It was followed by the results obtained from treatments containing 2, 1, and 0.5% of PEG 6000 concentration, respectively. The minimum percentage of shooting (~82.5%), mean shoots length (4.1 cm), mean number of nodes (4.4), mean number of leaves (13.1), and fresh weight (FW) of shoots (0.16 g) were shown by the MS medium lacking PEG 6000, i.e., the control treatment.

### 2.2. Estimation of Secondary Metabolites Production

**Figure 1:**
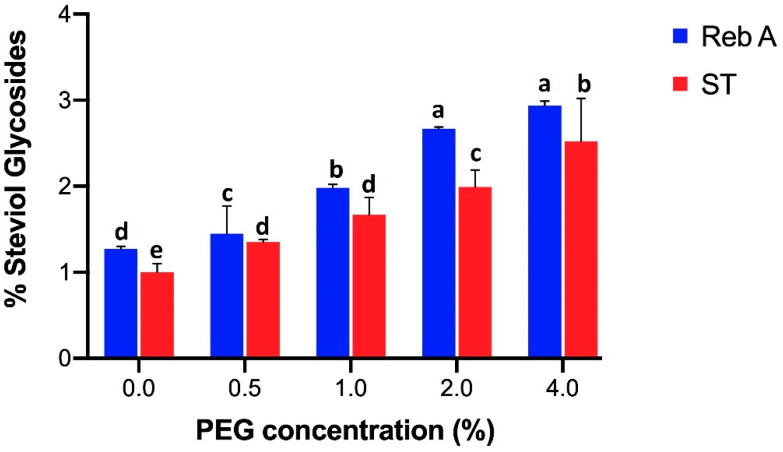
Effect of different PEG 6000 concentrations (0, 0.5, 1, 2, 4%) on shoots regarding rebaudioside A content represented with blue bars and stevioside content with red bars. For each parameter, bars with different case letters a–e are significantly different at the confidence interval level of 95% (Duncan’s multiple range test).

Figure 1 shows that the content of SGs, i.e., Reb A and ST illustrated by HPLC were found highest (Reb A 2.94% and ST 2.52%) under 4% of PEG 6000 stress. However, the PEG-deficient treatment revealed lowest amount of SGs (Reb A 1.27% and ST 1%).

**Figure 2:**
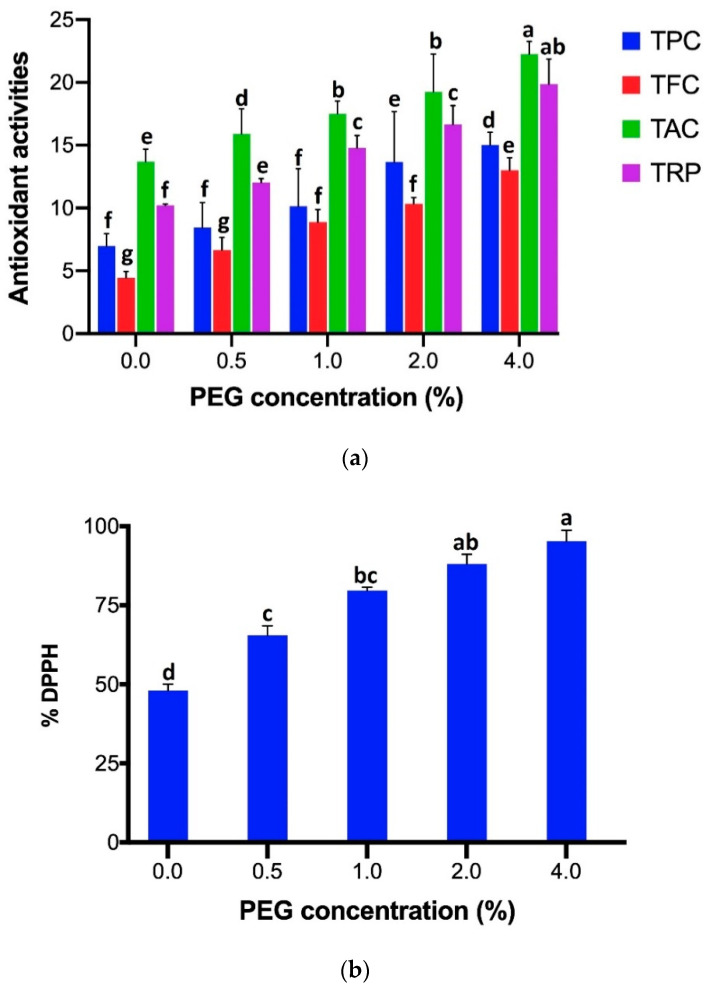
(**a**) Comparison of antioxidant activities (TPC, TFC, TAC, TRP) results expression for each assay of shoots grown under different PEG 6000 concentrations (0, 0.5, 1, 2, 4%). For each parameter, bars with different case letters are significantly different at the confidence interval level of 95% (Duncan’s multiple range test). (**b**) Comparison of % DPPH-free radical scavenging activity of shoots grown under different PEG 6000 concentrations (0, 0.5, 1, 2, 4%). Bars with different case letters a–g are significantly different at the confidence interval level of 95% (Duncan’s multiple range test).

In case of different nonenzymatic antioxidant activities such as TPC, TFC, TAC, TRP, and DPPH-FRSA, Figure 2a,b reveals that significantly highest content (TPC 15.03 µg/mg, TFC 13.01 µg/mg, TAC 22.26 µg/mg, TRP 19.87 µg/mg, and DPPH-FRSA 95.27%) was estimated by the shoots grown in MS medium augmented with 4% of PEG 6000 stress. It was followed by the amounts obtained under the stress of 2% of PEG 6000 and later, 1% of PEG 6000 stress, followed by the PEG 6000 stress of 0.5%. The least quantity of antioxidant activities (TPC 6.97 µg/mg, TFC 4.44 µg/mg, TAC 13.7 µg/mg, TRP 10.22 µg/mg, and DPPH-FRSA 48.03%) was elucidated by the shoots grown in PEG 6000-deficit medium.

## 3. Discussion

The current study outlines that differences in physiological characteristics, bioactive SGs content, and nonenzymatic antioxidant activities occur under the different concentrations of PEG 6000 stress; 0.5, 1, 1.5, 2, and 4%. A significant increment of all these parameters has been observed from 0.5 to 4% of PEG 6000 concentration. Few previous reports have enlightened the enhancement of growth and antioxidants after exposure of *S. rebaudiana* to PEG 6000 while others disagree on the growth and secondary metabolites increase and ultimately susceptibility to water deficiency in the environment [18,19,20,21,22,23,24]. Hajihashemi and Ehsanpour [21] documented an increase in antioxidant activities of *S. rebaudiana* under rising levels of PEG 6000 concentration, i.e., 0, 2, 4, 6%. Moreover, Hajihashemi and Sofo [20] reported reduction in plant growth but elevated levels of enzymatic and nonenzymatic antioxidants in this sweet herb under 5, 10, and 15% of PEG 6000 concentration. Hajihashemi et al. [20] also elucidated enhancement of antioxidant activities in the callus of *S. rebaudiana* treated with PEG 6000 (0 and 4%) along with different growth regulators. Additionally, Gorzi et al. [24] documented improved seedling growth and uprising antioxidant capacity upon exposure to PEG 6000 stress. These reports coincide with our study and prove the drought tolerance in *S. rebaudiana* on increasing PEG 6000 levels. On the contrary, Badran et al. [19] reported negative response of PEG 6000 stress on the ST production and positive response on shooting of *S. rebaudiana*. The plant shoots were exposed to different levels of PEG 6000, i.e., 0, 10, 20, 30 g/L in their study and the ultimate result of exposure was marked decrease of stevioside with rising PEG 6000 concentration. The more recent studies by Bogado and Nakayama [23] and Pradhan et al. [25] reported that PEG 6000 induced a negative effect on the in vitro growth, whereas Hajihashemi and Geuns [18] demonstrated negative influence of PEG 6000 on SGs of *S. rebaudiana*. The later exposed *Stevia* to 5, 10, and 15% PEG 6000 stress under greenhouse conditions and obtained results that are contradictory to our findings.

It is speculated that when drought stress is caused by water deficiency at critical levels, it manipulates the physiology as well as biochemistry of plants. In case of in vitro culture, *S. rebaudiana* plant cells imposed by PEG 6000 stress stimuli elicit significant alterations in the cellular environment. It involves activation of cellular receptors initiating signal transduction cascade that affects physiological processes. Changes in physiology influence primary metabolism that in turn causes signalling of secondary metabolism by providing biosynthetic precursors or intermediates. Hence, enhancement of biomass and bioactive components’ biosynthesis takes place simultaneously as shown by the current study. The possible mechanism involves cellular dehydration caused by PEG 6000 stress and resultant increased formation of ROS. Multiple cellular components like DNA, RNA, proteins, and lipids get damaged after excessive oxidation. Although ROS are produced even in the absence of stress and get scavenged by naturally occurring antioxidant, the stress conditions cause imbalance of ROS and antioxidants produced in cross-protection. ROS are signalling moieties that play a significant role in the controlled response against stress and programmed cell death. The defensive response is created under appropriate concentration of stress elicitor whereas concentration above threshold causes death of cells [26,27,28].

The tools of molecular biology such as transcriptomic and metabolic technologies have been employed for the determination of stress associated genes and elucidation of signalling pathways. It has been speculated that the transcription factors responsible for regulation of gene expression of biosynthesis of secondary metabolites get activated in response to PEG 6000-associated drought stress [18,29]. Hence, tolerance produced by tolerance-associated molecules is implicated on cellular level that regulates different steps of signal transduction system. Despite recent advancement, extensive research is needed in this domain to elucidate the effects of drought stress on the secondary metabolites’ formation and growth dynamics of the *S. rebaudiana* plant raised in vitro. Additionally, our current study should be applied on industrial scale to get enhanced content of desired pharmaceutical compounds by tissue culture.

## 4. Experimental Procedure

### 4.1. MS Medium Preparation and Shoot Organogenesis Conditions

The Murashige and Skoog (MS) culture medium [30] was prepared using 3% (*w*/*v*) sucrose. In order to study organogenesis, 5 polyethylene glycol (PEG 6000) treatments, i.e., 0, 0.5, 1, 2, 4% were prepared. pH of medium was adjusted to 5.7–5.8, and 0.8% (*w*/*v*) plant agar was added. The sterilization of medium was performed using autoclave at the pressure of 1.06 kg cm^−2^ and temperature of 121 °C for 15 min.

The *S. rebaudiana* seeds purchased from Shenyang Agricultural University, China were first disinfected with 0.1% (*w*/*v*) of mercuric (II) chloride (HgCl_2_). Then the culturing of these seeds was carried out on plain MS medium. The axillary shoot nodes excised from 4-week-old seedlings were incubated in various treatments of culture media. The growth room chamber having 16 h light/8 h dark photoperiod, 35 μmol m^−1^ s^−1^ irradiance, 24 ± 1 °C temperature, and 55–60% rate of relative humidity was utilized for conductance of experiment in triplicate. 15 explants per treatment were cultivated for 4 weeks. Various parameters of physiology, i.e., percentage (%) of nodal explants shooting, mean shoots length, mean number of leaves per regenerated shoot, mean number of nodes per explant, and fresh weight (FW) of shoots per explant were observed.

### 4.2. Extract Preparation for Steviol Glycosides Analysis

The leaves of in vitro regenerated shoots from each treatment were utilized for extraction of steviol glycosides (SGs). After careful washing of all shoots with sterile distilled water, their soaking was done on filter paper. Then, the plant material was dried at 60 °C for 48 h in an oven. The SGs were analysed by high performance liquid chromatography (HPLC) technique using Ultimate 3000 (Thermo Fisher, Temecula, CA, USA).

For HPLC, about 20 mg of each sample treatment was added to microcentrifuge tube containing 1 mL of 70% (*v*/*v*) methanol. This solution was incubated in an ultrasonic bath at 55 °C for 15 min, and then centrifuged at 12,000 rpm and 25 °C for 10 min. The supernatant was filtered using 0.22 µm PTFE Millipore syringe filters and later on, HPLC analysis was done by running all of samples in triplicate.

HPLC analysis was performed using an autosampler (WPS-3000-SL Dionex Semi Prep Autosampler) injecting 10 µL of each sample, a binary pump (LPG 3400SD Dionex) solvent delivery system working at a flow rate of 0.8 mL min^−1^, the column, Inertsil^®^ ODS-3 (GL Sciences Inc. Tokyo, Japan) with 150 × 4.6 mm in length and 5 μm particle size that was kept warm at 40 °C in a column oven system (TCC-3000SD Dionex, Watertown, MA, USA), and a dual wavelength absorbance detector operating at 210 and 350 nm (MWD-3100 Dionex UV-VIS Detector, Watertown, MA, USA). Finally, isocratic flow was performed using acetonitrile and 1% (*w*/*v*) phosphoric acid buffer mixture (68:32) for 20 min.

Pure SGs (100 mg L^−1^) were spiked on the final plant extracts and the percentage of recovery was measured from two individual extractions and three analytical HPLC runs of each extract. The confirmation of SGs was done using a fraction collector system (AFC-3000 UltiMate Fraction Collector; Thermo-Fisher Scientific, Waltham, MA, USA) as well as by using a thin-layer chromatography system. The contents of SGs, i.e., Reb A and ST were determined on the basis of their molar absorption at 210 nm in which Reb A was also utilized for calibration purpose. The standard SGs were purchased from ChromaDex^®^ (Irvine, CA, USA).

### 4.3. Extract Preparation for Antioxidant Activities Analysis

In order to perform different antioxidant activities, the *S. rebaudiana* leaf extracts were prepared by drying the leaves from different treatments at 100 °C in drying oven, and then taking 0.1 g of their fine powder that was dissolved in 500 μL of dimethyl sulfoxide (DMSO). It was vortexed for 5 min and then sonicated for 30 min. Centrifugation was performed at 10,000 rpm for 15 min and the supernatant was stored for performing antioxidant activities.

#### 4.3.1. Total Phenolic Content (TPC) and Total Flavonoid Content (TFC)

In leaf extracts of *S. rebaudiana*, total phenol content (TPC) was determined by performing the method of Khan et al. [31]. An aliquot of 20 µL (4 mg/mL) of DMSO stock solution of each sample was added in the respective well of 96 well plate. Then, 90 µL of Folin–Ciocalteu reagent was added in it and the plate was kept for 5 min. Later, 90 µL of sodium carbonate was added to the reaction mixture of all samples that were run in triplicate. The absorbance of samples was obtained at 630 nm using microplate reader. The standard used was gallic acid and the results were expressed as µg gallic acid equivalent per mg (µg GAE/mg).

Total flavonoid content (TFC) was determined by following the method of Khan et al. [31]. An aliquot of 20 µL (4 mg/mL) DMSO stock solution of each sample was added to the respective well of 96 well plate. Then, 10 µL of 1.0 M potassium acetate, 10 µL of 10% aluminium chloride, and 160 µL of distilled water were added to it and the mixture was kept at room temperature for 30 min. The absorbance of samples run in triplicate was measured at 630 nm using microplate reader. The standard used was quercetin and the results were expressed as µg quercetin equivalent per mg (µg QE/mg).

#### 4.3.2. Total Antioxidant Capacity (TAC) and Total Reducing Power (TRP)

Total antioxidant capacity (TAC) was determined by the method of Khan et al. [31]. An aliquot of 100 μL from stock solution of each sample (4 mg/mL in DMSO) was added to 96-well plate and 900 µL of reagent solutions containing 4 mM ammonium molybdate, 0.6 M sulfuric acid, and 28 mM sodium phosphate were mixed in it. The solution was incubated at 95 °C for 90 min and then cooled at room temperature. The absorbance of all samples run in triplicate was measured at 695 nm using microplate reader. Ascorbic acid was used as standard and the results were expressed as µg ascorbic acid equivalent per mg (µg AA/mg).

Total reducing power (TRP) was determined by following the method of Khan et al. [31]. An aliquot of 100 µL of each sample (4 mg/mL in DMSO) was added to 96-well plate and then 200 µL of phosphate buffer (0.2 M, pH 6.6) and 250 µL of 1% *w*/*v* potassium ferricyanide were added to it. This mixture was incubated at 50 °C for 20 min and later acidified with 200 µL of 10% (*w*/*v*) trichloroacetic acid. Centrifugation was performed at 3000 rpm for 10 min and the supernatant (150 µL) obtained was mixed with 50 µL of 0.1% (*w*/*v*) ferric chloride solution. The absorbance of all samples run in triplicate was measured at 630 nm. The standard used was ascorbic acid and results were expressed as µg ascorbic acid equivalent per mg (µg AA/mg).

#### 4.3.3. DPPH-Free Radical Scavenging Activity

2,2-diphenyl-1-picryl hydrazyl (DPPH)-free radical scavenging activity (FRSA) activity was performed according to the method of Khan et al. [31]. An aliquot of 10 µL (4 mg/mL) of *S. rebaudiana* leaf extracts were added to 96-well plate. 190 µL of DPPH [0.004% (*w*/*v*) in methanol] was added in it. This solution was incubated in darkness for 1 h. The absorbance of all samples run in triplicate was observed at 515 nm using microplate reader. Ascorbic acid was used as positive control while DMSO was used as negative control.

(1) $$\%\;\mathrm{inhibition}\;\mathrm{of}\;\mathrm{test}\;\mathrm{sample}=\;\%\;\mathrm{scavenging}\;\mathrm{activity}\;=\;\left(1-A_{\mathrm{bs}}/A_{\mathrm{bc}}\right)\;\times \;100$$

where A_bs_ indicates the absorbance of DPPH with sample and A_bc_ is the absorbance of only DPPH. The IC_50_ was calculated by using Table curve software 2D Ver. 4 (SYSTAT, San Jose, CL, USA).

### 4.4. Statistical Analysis

The design of experiments was randomized, and the statistical analysis of data was performed using SPSS, Version 17.0 (SPSS Inc., Chicago, IL, USA). Statistical difference was determined using ANOVA and the significance of difference between means ± SE (standard error) values was obtained using Duncan’s multiple range tests performed at *p* < 0.05.

## 5. Conclusions

This is the first report that brings to the spotlight how the major steviol glycosides (rebaudioside A and stevioside) respond when they get exposed to the abiotic stress induced by polyethylene glycol (PEG) 6000. Different growth parameters including percentage of explants shooting, mean shoots length, mean number of nodes, mean number of leaves, and fresh weight of shoots and antioxidant activities involving total phenolic content, total flavonoid content, total antioxidant capacity, total reducing power, and DPPH-free radical scavenging activity are also explored. Out of all concentrations, 4% of PEG concentration has been proved very efficacious in increasing the shoot organogenesis as well as the content of secondary metabolites in shoots of *S. rebaudiana* grown via tissue culture technique. Hence, this drought stress elicitor should be involved in further studies for significant enhancement and sustainable production of rebaudioside A and stevioside on commercial scale. Furthermore, signalling mechanisms of metabolic action of phenolic, flavonoid, and bioactive compounds produced by *S. rebaudiana* produced under drought stress should be finely investigated to unveil the ultimate reason for the described phenomenon.

## Figures and Tables

**Table 1:** Comparison of physiological parameters in 4-week-old shoots produced from nodal explants on Murashige and Skoog (MS) medium supplemented with different concentrations of PEG 6000.

Conc. of PEG (%)	Nodal Explants Shooting (%)	Mean Shoot Length (cm)	Mean No. of Nodes Per Explant	Mean No. of Leaves Per Regenerated Shoot	FW of Shoots Per Explant (g)
0	82.5	4.1 ± 0.01 ^e^	4.4 ± 0.01 ^e^	13.1 ± 0.01 ^c^	0.16 ± 0.01 ^d^
0.5	84.7	4.3 ± 0.01 ^d^	4.6 ± 0.01 ^d^	13.6 ± 0.02 ^c^	0.24 ± 0.01 ^c^
1.0	86.4	4.5 ± 0.01 ^c^	4.8 ± 0.01 ^c^	14.8 ± 0.01 ^b^	0.38 ± 0.01 ^b^
2.0	88.9	4.7 ± 0.02 ^b^	5.0 ± 0.01 ^b^	16.5 ± 0.02 ^a^	0.45 ± 0.01 ^a^
4.0	92.4	5.1 ± 0.01 ^a^	5.3 ± 0.01 ^a^	17.9 ± 0.01 ^a^	0.52 ± 0.01 ^a^

±: standard error, small alphabetical letters ^a–e^ with mean values representing the difference among applied PEG concentration within the columns according to Duncan’s multiple range test at confidence level of 95%.

## Abbreviations

MS, Murashige and Skoog; Reb A, rebaudioside A; ST, stevioside; DPPH, 2,2-diphenyl-1-picryl hydrazyl; HPLC, high performance liquid chromatography; TPC, total phenolic content; TFC, total flavonoid content; TAC, total antioxidant capacity; TRP, total reducing power; DMSO, dimethyl sulphoxide; ROS, reactive oxygen species.
